# Identifying county characteristics associated with resident well-being: A population based study

**DOI:** 10.1371/journal.pone.0196720

**Published:** 2018-05-23

**Authors:** Brita Roy, Carley Riley, Jeph Herrin, Erica S. Spatz, Anita Arora, Kenneth P. Kell, John Welsh, Elizabeth Y. Rula, Harlan M. Krumholz

**Affiliations:** 1 Department of Internal Medicine, Section of General Internal Medicine, Yale School of Medicine, New Haven, Connecticut, United States of America; 2 Department of Pediatrics, University of Cincinnati College of Medicine, Cincinnati, Ohio, United States of America; 3 Division of Critical Care, Cincinnati Children’s Hospital Medical Center, Cincinnati, Ohio, United States of America; 4 Department of Internal Medicine, Section of Cardiovascular Medicine, Yale School of Medicine, Center for Outcomes Research and Evaluation, New Haven, Connecticut, United States of America; 5 Department of Internal Medicine, Section of General Internal Medicine, Yale School of Medicine, New Haven, Connecticut, United States of America; 6 Tivity Health, Franklin, Tennessee, United States of America; 7 Yale University, New Haven, Connecticut, United States of America; 8 Department of Internal Medicine, Section of Cardiovascular Medicine, Yale School of Medicine; Department of Health Policy and Management, Yale School of Public Health; Center for Outcomes Research and Evaluation, Yale-New Haven Hospital, New Haven, Connecticut, United States of America; University of Texas Medical Branch at Galveston, UNITED STATES

## Abstract

**Background:**

Well-being is a positively-framed, holistic assessment of health and quality of life that is associated with longevity and better health outcomes. We aimed to identify county attributes that are independently associated with a comprehensive, multi-dimensional assessment of individual well-being.

**Methods:**

We performed a cross-sectional study examining associations between 77 pre-specified county attributes and a multi-dimensional assessment of individual US residents’ well-being, captured by the Gallup-Sharecare Well-Being Index. Our cohort included 338,846 survey participants, randomly sampled from 3,118 US counties or county equivalents.

**Findings:**

We identified twelve county-level factors that were independently associated with individual well-being scores. Together, these twelve factors explained 91% of the variance in individual well-being scores, and they represent four conceptually distinct categories: demographic (% black); social and economic (child poverty, education level [<high school, high school diploma/equivalent, college degree], household income, % divorced); clinical care (% eligible women obtaining mammography, preventable hospital stays per 100,000, number of federally qualified health centers); and physical environment (% commuting by bicycle and by public transit).

**Conclusions:**

Twelve factors across social and economic, clinical care, and physical environmental county-level factors explained the majority of variation in resident well-being.

## Introduction

Well-being is defined as “a person’s cognitive and affective evaluations of his or her life,” and includes “emotional reactions to events as well as cognitive judgements of satisfaction and fulfillment.”[1] It is a holistic, positively framed assessment of health and quality of life that captures aspects of an individual’s physical, mental, and social health, beyond the presence or absence of disease, as well as an introspective evaluation of one’s life.[2–6] Higher well-being by definition has inherent positive value, and an individual’s well-being has been independently associated in a dose-response manner with lower risk of cardiovascular events and greater longevity.[7–12] Recently, our group extended these findings by reporting a relationship between aggregate well-being and life expectancy at the county level, after accounting for poverty, education and race.[13] Given the positive value of well-being and the recent focus on population health, there is interest in designing communities that support this construct,[14–16] but more evidence is needed about local factors that are associated with population well-being.

Where one lives may be an important determinant of a person’s well-being, and indeed, well-being varies by region.[17] Community attributes such as access to basic healthcare and social services, safe and clean streets, public transportation, green spaces and healthy foods, along with social aspects such as neighborhood cohesion and trust, may influence peoples’ evaluation of their own well-being.[18] Many of these community attributes are modifiable, and thus are increasingly recognized as a focus for improving a population’s health and well-being. In fact, the Centers for Medicare and Medicaid Services have begun to pilot accountable health community models.[19] These new payment models acknowledge that social and community factors influence health outcomes, and compensation to support positive modification of these factors is needed. Although several community factors have been associated with well-being or aspects of well-being, knowing which of these factors are most strongly associated with health and well-being is important yet challenging because many of these attributes are correlated with each other. As such, it is necessary to identify community attributes that are strongly and independently associated with better health outcomes and higher well-being. Identifying these attributes could provide evidence for prioritization of community-level targets for interventions that aim to promote well-being.

We used a systematic method[20] to identify independent associations with well-being among highly interrelated county characteristics drawn from multiple community sectors emphasized by established theoretical frameworks.[15, 21–25] We performed a cross-sectional study using data from the Gallup-Sharecare Well-Being Index (WBI), a comprehensive, multi-dimensional well-being assessment of over 350,000 Americans annually. We demonstrated independent associations among individual well-being scores and 77 county factors related to the following community sectors: demographics, social and economic status, clinical care, health behaviors, and the physical environment. To compare our results with the emerging literature related to life satisfaction, we also conducted an exploratory analysis of the relationship between county factors and life evaluation, a component of the WBI consisting of a subjective overall assessment of one’s current and five-year outlook on life.[26, 27]

## Materials and methods

### Measures: Well-Being

We used WBI data from January 1, 2010 to December 31, 2012 to assess well-being. Gallup-Sharecare conducted between 500 to 1000 telephone surveys with a random sample each day, 350 days per year. The sample included adults aged 18 years and older residing in the United States, who spoke either English or Spanish and have either a landline or cellular phone. Gallup-Sharecare used a structured sampling design to obtain data from all 50 states and the District of Columbia.[28]

The WBI was developed based on the work of psychology experts.[4, 29] Briefly, survey items that aligned with previous research on well-being were initially compiled by experts in the field.[30, 31] Based on reviews of the literature, items were selected to encompass both hedonic well-being (i.e. , people’s feelings and thoughts about their lives) [32] and eudemonic well-being (i.e., an individual’s judgments about the meaning and purpose in one’s life),[11] and thus incorporated items assessing daily emotional experience and a wide variety of evaluative domains, such as overall life, standard of living, and satisfaction with community, work, relationships, and personal health. Factor analysis using data from a large, representative national sample was then used to determine the final set of questions. Criterion validity of regionally aggregated data was established by examining correlations with regional health and socioeconomic indicators.[33] Subsequently, principal component analyses and confirmatory factor analyses were used to create an instrument valid for measuring well-being at the individual level. The individual well-being measure has acceptable reliability, internal and external validity.[34] It includes 40 self-reported items organized into six domains representing key aspects of well-being that are similar to other multi-dimensional constructs of well-being[30] (S1 Table): life evaluation, emotional health, work environment, physical health, healthy behaviors, and basic access.[28] Our primary outcome was the composite individual well-being score (iWBS), which is the unweighted mean of the six domain scores, each scaled to range from 0–100.

Our secondary outcome was the life evaluation index (LEI). The LEI is a two-item measure adapted from Cantril’s Self-Anchoring Striving Scale,[26] a reflection on one’s overall experience of life and future outlook. These items require respondents to rate on a scale from 0 to 10 their overall current life situation as well as their expected life situation in five years. The LEI is calculated by averaging the number of points (e.g., where the participant places themselves on the ladder) from both items and multiplying by ten, to match the scale of the other domains with a maximum score of 100.

### Measures: County attributes

We used U.S. county or county equivalent (e.g., boroughs, towns, or parishes, depending on the state) as our unit of analysis because it is a defined unit for community initiatives and policy change. County was the smallest geographic unit for which we could both assign respondents and identify a large range of local attributes.

Existing theoretical frameworks agree that a variety of community properties affect mortality, health-related quality of life, and subjective health and well-being.[35] [36] [22, 23, 37, 38] After reviewing multiple theoretical frameworks, we adapted and expanded the University of Wisconsin Population Health Institute County Health Rankings and Roadmaps (CHRR)[39] framework to encompass the major themes of categories of county factors theorized to be associated with health and well-being across all frameworks. These included social and economic, health behaviors, clinical care, physical environment, demographic, and psychosocial categories of county factors. We identified, *a priori*, 114 county factors within these categories that we postulated would influence the six domains of resident well-being in our conceptual model (S1 Fig).

We then searched for zip code or county level sources for data on these 114 pre-specified county factors hypothesized to be associated with resident well-being. We first drew factors from the Robert Wood Johnson Foundation County Health Rankings (years 2010, 2012 2013), which aggregates data from multiple sources. We captured data on additional factors from the Area Health Resource File (2007–2012), American Community Survey/US Census (2010 and 2012), and Nielsen Pop-Facts (2013). We were able to obtain data on 77 county factors within 5 categories that were well-aligned with the pre-specified factors (Table 1). No consistent county or zip code level data were available across years 2010–2013 for the other 37 factors (S1 Fig), which included all factors in the psychosocial category, so this category was removed from our model. Continuous variables were categorized into quintiles by county, unless highly skewed (i.e., more than 20% of counties having the same value), in which case two observers inspected the distribution and agreed on reasonable categories. All data were merged with the WBI participant data using the Federal Information Processing Standard code.

**Table 1:** Mean resident composite well-being scores across quintiles of 77 county characteristics.

County Characteristics			Q1	Q2	Q3	Q4	Q5	P-value	R^2^
**Demographic Factors**									
% English Only Spoken at Home			74.3	74.1	73.1	72.5	70.4	<0.001	0.29
% Female									
	Total		72.8	74.1	73.8	74.0	73.6	<0.001	0.02
	< = 15 years		73.8	73.6	73.8	73.7	73.9	0.006	0.01
	< = 19 years		73.3	73.2	73.6	74.1	74.0	<0.001	0.07
	< = 24 years		74.6	73.7	73.3	73.4	73.9	<0.001	0.07
	< = 44 years		73.5	73.2	72.9	73.3	74.2	<0.001	0.09
	< = 64 years		74.0	73.8	73.4	73.6	73.9	<0.001	0.03
	65+ years		74.4	73.6	73.3	72.3	73.5	<0.001	0.15
% Moved from a Different County in past 5y			73.4	73.7	73.8	74.1	74.2	<0.001	0.05
Population Density			73.3	72.6	72.2	73.0	74.3	<0.001	0.17
Race									
	% Asian (.2, .3–1, 1–2, >2)		71.4	73.1	73.8	74.6	—	<0.001	0.64
	% Black (< .5, .5–2, 2–10, 10–30, >30)		73.1	73.8	74.0	73.8	73.1	0.005	0.54
	% White		73.9	74.0	73.8	73.3	72.1	<0.001	0.05
Retirement Destination (No, Yes)			73.7	74.0	—	—	—	0.003	0.54
% Rural (≤5, 6–30)			74.1	71.9	—	—	—	<0.001	0.59
% Urban			71.7	71.6	72.3	73.4	74.3	<0.001	0.27
**Social and Economic Factors**									
Education									
	% Less Than 9th Grade		74.6	74.0	73.5	73.5	72.4	<0.001	0.26
	% 9th to 12th Grade, No Diploma		75.4	74.3	73.2	72.1	70.5	<0.001	0.69
	% High School Graduate or Equivalent		74.6	73.3	72.5	72.2	71.4	<0.001	0.41
	% Some College, No Degree		73.7	73.7	73.9	73.7	73.8	<0.001	0.00
	% Associate's Degree		72.9	73.6	73.9	73.9	74.1	<0.001	0.08
	% Bachelor's Degree		69.6	71.6	72.5	73.4	74.8	<0.001	0.69
	% Graduate or Professional Degree		70.8	71.4	72.0	73.3	74.7	<0.001	0.56
GINI Coefficient			74.0	74.0	73.3	73.9	73.7	<0.001	0.02
Marital Status									
	% Never Married		72.9	72.9	73.5	74.0	74.0	<0.001	0.05
	% Divorced		74.7	74.1	73.7	73.0	72.2	<0.001	0.25
Mean Household Income			71.3	71.8	72.6	73.8	74.8	<0.001	0.51
Median Household Size			74.0	73.5	73.4	73.5	74.2	<0.001	0.06
Poverty									
	% Children in Poverty		75.2	73.9	73.4	72.5	71.2	<0.001	0.53
	% Persons in Poverty		75.1	74.3	73.5	72.9	71.6	<0.001	0.41
% School Enrollment									
	Nursery School, Preschool		73.1	73.6	73.7	74.2	74.0	<0.001	0.02
	Kindergarten		74.0	74.0	74.0	73.3	72.1	<0.001	0.07
% Single Parent Households			75.1	74.3	73.9	73.5	73.0	<0.001	0.10
% Unemployed			74.9	74.4	73.8	73.2	72.3	<0.001	0.24
Violent Crime Rate			74.3	74.0	74.1	74.0	73.5	0.054	0.18
**Clinical Care Factors**									
% Diabetes with HbA1c Test			72.9	73.5	73.8	74.1	74.3	<0.001	0.09
ED Visits/100k			72.4	74.5	74.2	73.5	72.6	<0.001	0.13
# Federally Qualified Health Centers (0,1,2+)			73.5	73.4	73.9	—	—	<0.001	0.54
Healthcare Practitioners									
	Dentists/100k		71.1	72.1	73.0	73.5	74.6	<0.001	0.32
	GPs/100k		72.3	73.3	73.9	73.9	74.4	<0.001	0.10
	GPs/Specialists		74.2	73.5	72.6	72.3	72.6	<0.001	0.12
% Health Spending			74.7	73.5	72.9	71.9	71.6	<0.001	0.42
Acute and Long Term Care Capacity									
	# Hospitals (0, 1, 2, 3–4, 5–10, 11+)	72.4	72.8	73.6	73.9	74.3	74.2	<0.001	0.56
	# Hospital Beds		72.4	72.6	72.5	73.2	74.1	<0.001	0.10
	# NH Beds (0, 50, 51–100, 101+)		73.5	74.1	73.9	74.1	—	<0.001	0.54
	# Psych Hospitals (0, 1, 2+)		73.5	74.1	74.2	—	—	<0.001	0.55
% Mammography			72.1	72.8	73.5	74.3	74.7	<0.001	0.27
% Medicaid			75.3	74.2	73.5	73.1	72.3	<0.001	0.41
% Prescription Drug Spending			74.7	73.7	72.9	71.8	71.4	<0.001	0.42
Preventable Hospital Stays			74.9	74.1	73.2	72.1	70.1	<0.001	0.49
Health Professions Schools									
	# DDS Schools (0, 1+)		73.7	74.0	—	—	—	0.002	0.54
	# DO Schools (0, 1+)		73.8	73.6	—	—	—	0.732	0.54
	# MD Schools (0, 1+)		73.6	74.3	—	—	—	<0.001	0.55
	# Optometry Schools (0, 1+)		73.7	74.4	—	—	—	0.049	0.54
	# Pharmacy Schools (0, 1+)		73.6	74.2	—	—	—	<0.001	0.55
	# RN Schools with BSN Program (0, 1+)		73.4	74.1	—	—	—	<0.001	0.55
Short Term General Hospitals Utilization Rate									
	00–39% (0,1–2,2)		73.7	73.9	74.2	—	—	0.433	0.54
	40–59% (0,1–2,2)		73.5	73.9	74.0	—	—	<0.001	0.54
	60–79% (0,1–2,2)		73.0	73.9	74.2	—	—	<0.001	0.56
	80+% (0,1–2,2)		73.5	74.2	74.2	—	—	<0.001	0.55
% Uninsured Adults			74.0	73.5	73.8	73.8	73.7	<0.001	0.02
**Physical Environment Factors**									
% Commute by									
	Bicycle (0, ≤0.1, .1–1, >1)		71.3	73.2	74.0	75.0	—	<0.001	0.61
	Car, Truck, or Van		74.7	74.3	73.5	73.0	71.7	<0.001	0.29
	Public transit (0, ≤0.5, .5–1, 1–3, >3)		71.8	72.8	73.6	74.2	74.5	<0.001	0.59
	Walk (≤1, 1–3, 3–5, 5–10, >10)		73.1	73.7	74.1	74.0	74.9	<0.001	0.55
	Work at Home (≤2, 2–4, 4–8, 8–10, >10)		70.9	73.1	74.6	74.9	74.6	<0.001	0.60
Daily Fine Particulate Matter			74.1	74.0	73.7	73.9	72.9	<0.001	0.07
Farming Community (No, Yes)			73.8	72.9	—	—	—	0.893	0.54
% Good Air Quality Days			74.0	74.4	73.9	74.0	73.6	0.011	0.34
Housing Unit Density per Square Mile			73.2	72.6	72.3	72.9	74.2	<0.001	0.16
Number of Nearby Toxic Waste Sites (0,1,2+)			73.4	74.0	74.7	—	—	<0.001	0.56
% Water Violation (1–5, 6–10, >10)			73.5	74.1	73.9	73.2	—	<0.001	0.55
**Health Behaviors Factors**									
% Food Out Spending			73.5	73.4	72.9	73.8	74.3	<0.001	0.09
% Fruit/Veg Spending			74.1	73.9	73.6	73.5	73.8	<0.001	0.05
% People with Limited Access to Health Foods			72.3	73.2	73.9	74.0	74.0	<0.001	0.05
Recreational Facilities/100k			71.4	72.1	73.2	74.0	74.9	<0.001	0.29
% Restaurants that Serve Fast Food			73.6	74.1	74.0	73.8	72.9	<0.001	0.05

Each factor was categorized by equally distributed quintiles, unless noted in parentheses. Bivariate associations for each county factor with resident well-being were tested and level of significance is noted by the Wald P-value. Abbreviations: HbA1c = Hemoglobin A1c; ED = Emergency department; GP = General practitioner; NH = Nursing home; RN = Registered nurse; BSN = Bachelor of science in nursing; DDS = Doctor of dental surgery; DO = Doctor of osteopathy; MD = Doctor of medicine

### Analysis

We initially performed descriptive analyses of community attributes across counties. Because we expected that many county factors would be correlated within and across categories (e.g., high percent of population uninsured may be associated with low income; low educational attainment may be associated with high crime rates), we used a sequential approach to identify independent factors within each category and then across all categories. First, we evaluated bivariate associations between each of the 77 county-level predictor variables with participants’ WBI scores using a mixed effects linear model with random effect for county and individual well-being as the dependent variable. For each model, we calculated the overall (Wald) P-value for the county attribute and the model R^2^ as the proportion of variance explained at the county level.[40] We eliminated variables that were not significantly associated with the iWBS (overall P-value >0.05) or did not explain at least 20% of the variance in the iWBS (R^2^<0.20). This last criterion was chosen based on the distribution of R^2^ values, which had a substantial gap below 20%. Of those variables retained, we assessed for multi-collinearity within each category using variance decomposition proportions and eliminated redundant measures: if the retained variables in a category had a singular value greater than 20, variance decomposition proportions for each variable were examined, and if two or more contributed more than 50%, we retained the one with the greatest value.[41] This approach has been used previously by health services researchers to reduce large numbers of related factors to a smaller representative set.[20, 42] We then estimated a single model for each category including factors retained from this process. Then, we estimated a combined model including all variables that were independently significant (p<0.05) in their respective category-specific model. Our final model included only variables that were independently significant in the combined model (p<0.05).

Secondary analyses were performed using the same sequential approach, but with the LEI as the outcome. We focused on this particular domain to separate findings from those related directly to health behaviors and outcomes, and to compare our results with prior studies that have used this measure or a similar assessment of overall life evaluation.[17]

Analyses were performed using Stata 14.1 (2015, StataCorp, College Station, TX). The Yale University Human Subjects Committee approved this study.

## Results

The cohort included 338,846 survey participants, representing 3,118 counties in our analysis. Overall, the mean (SD) iWBS was 73.8 (15.4) and mean LEI was 72.5 (18.8). Counties had a mean aggregate composite well-being score of 66.5 (SD 5.4), with a range of 39.9 to 90.6.

In bivariate analyses, 73 variables across all categories were significantly associated with iWBS (p<0.05; Table 1). In the demographic category, measures of race, including higher percent Black (p = 0.005; R2 = 0.54) and higher percent Asian (p<0.001; R^2^ = 0.64), as well as measures of area characteristics, including being a retirement destination (p = 0.003; R^2^ = 0.54) and being less rural (p<0.001; R^2^ = 0.59), were associated with higher iWBS, and each explained over half of the variance in iWBS. In the social and economic category, lower percent without a high school diploma and higher percent with a Bachelor’s degree were both associated with higher well-being (p<0.001), and these factors explained the greatest amount of variation in resident well-being (R^2^ = 0.69 for both). In the clinical care category, variables related to healthcare spending, including lower percent with Medicaid (p<0.001; R^2^ = 0.41) and lower percent income spent on prescription medications (p<0.001; R^2^ = 0.42) and on health care (p<0.001; R^2^ = 0.42), as well as variables related to healthcare capacity, including greater number of hospitals (p<0.001; R^2^ = 0.56), number of psychiatric hospitals (p<0.001; R^2^ = 0.55), number of federally qualified health centers (p<0.001; R^2^ = 0.54), and presence of medical (p<0.001; R^2^ = 0.55), dental (p = 0.002; R^2^ = 0.54), pharmacy (p<0.001; R^2^ = 0.55), and nursing schools (p<0.001; R^2^ = 0.54) were associated with higher iWBS scores and explained the greatest amount of variation in iWBS. In the physical environment category, higher percent commuting by bicycle (p<0.001; R^2^ = 0.61) or public transport (p<0.001; R^2^ = 0.59) or working from home (p<0.001; R2 = 0.60) were associated with higher iWBS scores and explained the greatest amount of variation in iWBS. In the health behavior category, number of recreational facilities per hundred thousand explained the greatest amount of variance in iWBS (p<0.001; R^2^ = 0.29).

Of the 73 variables significantly associated with iWBS, 40 explained greater than 20% of the variation in well-being (R^2^>0.20), and thus were retained for within-category multivariable analyses (Table 2). In these multivariable models, 14 variables were no longer independently associated with iWBS after accounting for the effect of other factors in the same category. The social and economic category explained the greatest amount of variance (R^2^ = 0.86) and the health behavior category explained the least amount of variance (R^2^ = 0.29) in iWBS.

**Table 2:** Category-specific models for iWBS.

County Characteristics		Q1	Q2	Q3	Q4	Q5	P-value	R^2^
**Demographic Factors**								0.493
% English Only Spoken at Home		ref	0.31	0.08	-0.12	-1.72	<0.001	
Race								
	% Asian (.2, .3–1, 1–2, >2)	ref	0.87	1.64	2.49	—	<0.001	
	% Black (< .5, .5–2, 2–10, 10–30, >30)	ref	-0.98	-1.68	-1.95	-1.77	<0.001	
Retirement Destination (No, Yes)		ref	0.42	—	—	—	0.002	
% Rural (≤5, 6–30)		ref	-0.26	—	—	—	0.277	
% Urban		ref	-0.08	0.14	0.44	0.74	0.071	
**Social and Economic Factors**								0.856
Education								
	% Less Than 9th Grade	ref	0.08	-0.03	0.13	-0.24	0.155	
	% 9th to 12th Grade, No Diploma	ref	-0.60	-1.12	-1.32	-1.80	<0.001	
	% High School Graduate or Equivalent	ref	-0.60	-0.73	-0.79	-1.13	<0.001	
	% Bachelor's Degree	ref	1.11	1.45	1.75	1.84	<0.001	
Marital Status								
	% Divorced	ref	-0.16	-0.44	-0.74	-0.99	<0.001	
Mean Household Income		ref	0.01	0.01	0.32	0.38	0.031	
Poverty								
	% Children in Poverty	ref	-0.36	-0.59	-0.72	-1.30	<0.001	
% Unemployed		ref	-0.15	-0.38	-0.30	-0.38	0.022	
**Clinical Care Factors**								0.797
# Federally Qualified Health Centers (0,1,2+)		ref	-0.08	-0.30	—	—	0.019	
Healthcare Practitioners								
	Dentists/100k	ref	0.39	0.51	0.62	0.95	<0.001	
Acute and Long Term Care Capacity								
	# Hospitals (0, 1, 2, 3–4, 5–10, 11+) ref	0.45	0.44	0.52	0.58	0.75	0.264	
	# NH Beds (0, 50, 51–100, 101+)	ref	0.10	0.27	0.08	—	0.390	
	# Psych Hospitals (0, 1, 2+)	ref	-0.04	0.14	—	—	0.692	
% Mammography		ref	0.05	0.32	0.70	1.04	<0.001	
% Medicaid		ref	-0.75	-1.45	-1.66	-2.14	<0.001	
% Prescription Drug Spending		ref	-0.47	-0.54	-1.22	-1.46	<0.001	
Preventable Hospital Stays		ref	-0.18	-0.82	-1.22	-2.15	<0.001	
Health Professions Schools								
# DDS Schools (0, 1+)		ref	-0.56	—	—	—	0.022	
# MD Schools (0, 1+)		ref	0.57	—	—	—	0.003	
# Optometry Schools (0, 1+)		ref	0.85	—	—	—	0.008	
# Pharmacy Schools (0, 1+)		ref	-0.14	—	—	—	0.422	
# RN Schools with BSN Program (0, 1+)		ref	-0.05	—	—	—	0.649	
Short Term General Hospitals Utilization Rate								
40–59% (0,1–2,2)		ref	-0.15	-0.39	—	—	0.106	
60–79% (0,1–2,2)		ref	-0.11	0.08	—	—	0.346	
80+% (0,1–2,2)		ref	-0.11	0.02	—	—	0.699	
**Physical Environment Factors**								0.643
% Commute by								
	Bicycle (0, <0.1, .1–1, >1)	ref	1.26	1.56	2.14	—	<0.001	
	Car, Truck, or Van	ref	-0.32	-0.28	-0.44	-0.82	0.310	
	Public transit (0, <0.5, .5–1, 1–3, >3)	ref	0.88	1.16	1.61	1.82	<0.001	
	Walk (<1, 1–3, 3–5, 5–10, >10)	ref	-0.39	-0.81	-0.92	-0.97	0.037	
	Work at Home (<2, 2–4, 4–8, 8–10, >10)	ref	1.07	2.03	2.40	1.91	<0.001	
% Good Air Quality Days		ref	0.39	0.04	0.45	0.29	0.052	
Number of Nearby Toxic Waste Sites (0,1,2+)		ref	0.23	0.21	—	—	0.203	
% Water Violation (1–5, 6–10, >10)		ref	-0.04	-0.02	-0.33	—	0.298	
**Health Behaviors Factors**								0.292
Recreational Facilities/100k		ref	0.23	1.00	1.89	2.92	<0.001	

Correlation coefficients for the association between each variable that was significantly associated with iWBS in bivariate analyses, independent of other variables within the same category. Each factor was categorized by equally distributed quintiles, unless noted in parentheses. P-value reported is the Wald P-value for trend across quintiles. R^2^ is the amount of variance in resident well-being explained by all factors within each category. Abbreviations: NH = Nursing home; RN = Registered nurse; BSN = Bachelor of science in nursing; DDS = Doctor of dental surgery; MD = Doctor of medicine

**Fig 1:**
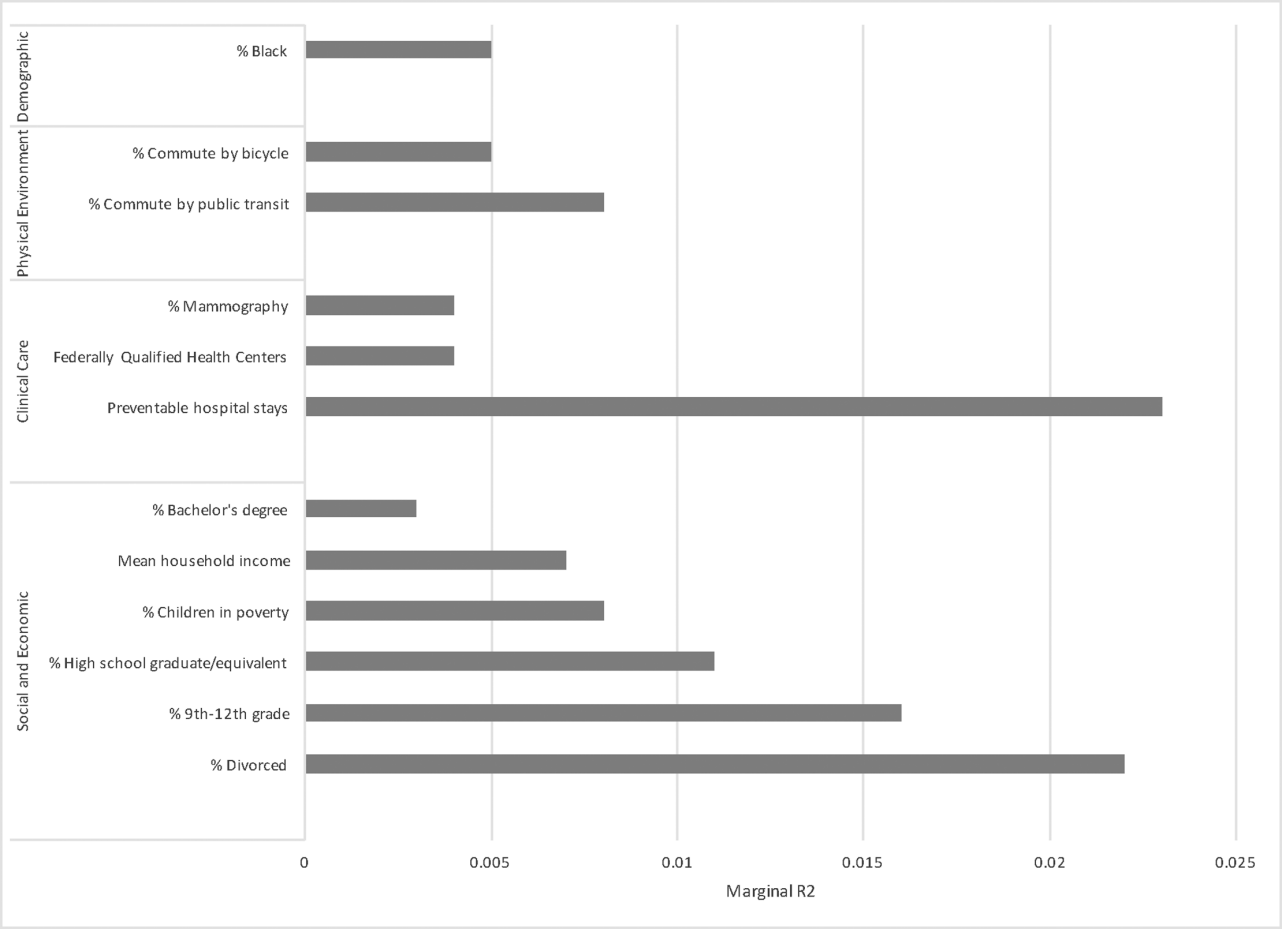
Marginal R2 of variables in iWBS final model. Comparison of the magnitude of reduction in county level variance explained if that variable was excluded from the model (marginal R^2^). Factors with larger marginal R^2^ contribute more to the final model than factors with a smaller marginal R^2^.

After dropping non-significant variables within each category model, 27 county factors remained and were included in a combined model assessing county factors with iWBS (Table 3, Combined Model). Of these, only 13 county factors remained significantly associated with iWBS after accounting for the effect of other variables in the model and were moved forward into the final model. In this final model, twelve variables, originally from four different categories, remained significantly associated with well-being (Table 3, Final Model). None of the variables from the health behavior category contributed independently to the final model. Together, these twelve variables explained 91% of the variance in iWBS. The three variables with the biggest effect on higher individual well-being, as measured by the magnitude of explained variance lost if the variable were omitted from the full model (Fig 1), were: lower rates of preventable hospital stays, lower percent divorced, and lower percent without high school diploma.

**Table 3:** Combined and final models of county characteristics independently associated with iWBS.

County Characteristics		Combined Model						Final Model					
		Q1	Q2	Q3	Q4	Q5	Wald P-value	Q1	Q2	Q3	Q4	Q5	Wald P-value
**Demographic Factors**													
% English Only Spoken at Home		ref	-0.22	-0.33	-0.37	-0.82	0.083						
Race													
% Asian (< .2, .3–1, 1–2, >2)		ref	-0.08	-0.35	-0.30	—	0.282						
% Black (< .5, .5–2, 2–10, 10–30, >30)		ref	0.28	0.15	0.11	0.93	<0.001	ref	-0.09	-0.06	0.04	0.81	<0.001
Retirement Destination (No, Yes)		ref	0.15	—	—	—	0.250						
% Urban		ref	0.22	0.46	0.79	0.91	0.111						
**Social and Economic Factors**													
Education													
	% 9^th^ to 12^th^ Grade, No Diploma	ref	-0.52	-0.97	-1.10	-1.84	<0.001	ref	-0.50	-0.94	-1.07	-1.54	<0.001
	% High School Graduate or Equivalent	ref	-0.42	-0.68	-0.38	-0.75	<0.001	ref	-0.53	-0.59	-0.56	-0.82	<0.001
	% Bachelor’s Degree	ref	1.39	1.43	1.63	1.79	0.001	ref	0.90	1.09	1.34	1.43	<0.001
Marital Status													
	% Divorced	ref	-0.02	-0.22	-0.74	-0.78	<0.001	ref	-0.30	-0.50	-0.84	-1.03	<0.001
Mean Household Income		ref	0.13	0.47	0.66	0.99	<0.001	ref	0.02	0.13	0.36	0.65	<0.001
Poverty													
	% Children in Poverty	ref	-0.28	-0.41	-0.34	-0.97	0.004	ref	-0.43	-0.64	-0.71	-1.36	<0.001
% Unemployed		ref	-0.21	-0.26	-0.28	-0.34	0.383						
**Clinical Care Factors**													
# Federally Qualified Health Centers (0,1,2+)		ref	-0.12	-0.32	—	—	0.028	ref	-0.05	-0.31	—	—	0.002
Healthcare Practitioners													
	Dentists/100k	ref	0.12	-0.13	-0.22	-0.41	0.151						
% Mammography		ref	-0.02	0.15	0.38	0.29	0.021	ref	-0.13	0.02	0.22	0.29	0.003
% Medicaid		ref	-0.02	-0.11	-0.05	0.02	0.903						
% Prescription Drug Spending		ref	-0.21	0.18	0.00	0.11	0.075						
Preventable Hospital Stays		ref	-0.16	-0.58	-0.71	-1.14	<0.001	ref	-0.21	-0.59	-0.73	-1.39	<0.001
Health Professions Schools													
# DDS Schools (0, 1+)		ref	-0.14	—	—	—	0.384						
# MD Schools (0, 1+)		ref	0.37	—	—	—	0.005	ref	0.13	—	—	—	0.278
# Optometry Schools (0, 1+)		ref	0.38	—	—	—	0.079						
**Physical Environment Factors**													
% Commute by													
	Bicycle (0, <0.1, .1–1, >1)	ref	0.26	0.58	0.89	—	<0.001	ref	0.10	0.36	0.79	—	<0.001
	Car, Truck, or Van	ref											
	Public transit (0, <0.5, .5–1, 1–3, >3)	ref	-0.01	-0.03	-0.10	-0.57	0.002	ref	0.08	-0.03	-0.05	-0.54	<0.001
	Walk (<1, 1–3, 3–5, 5–10, >10)	ref	0.04	0.10	0.04	0.08	0.983						
	Work at Home (<2, 2–4, 4–8, 8–10, >10)	ref	-0.11	0.11	0.24	0.35	0.236						
% Good Air Quality Days		ref	0.13	-0.03	0.27	0.32	0.081						
**Health Behaviors Factors**													
Recreational Facilities/100k		ref	-0.88	-0.76	-0.60	-0.58	0.203						

Combined model: independent correlation coefficients when including all variables that were independently significant in their relationship with iWBS in their respective category-specific model. Final model: independent correlation coefficient in a model that includes only variables significantly associated with WBI in combined model. Abbreviations: DDS = Doctor of dental surgery; MD = Doctor of medicine

In secondary bivariate analyses with LEI as the outcome, almost all county characteristics in all five categories were significantly associated with the life evaluation scores and 39 of these variables explained greater than 20% of the variation in the LEI, (S2 Table). None of the variables in the health behavior category explained greater than 20% variation in LEI, thus no category-specific model was performed for the health behavior category. The social and economic category explained the greatest amount of variance (R^2^ = 0.67), the demographic and physical environment categories explained a similar amount of variance (R^2^ = 0.55 and 0.54, respectively), and the clinical care category explained the least amount of variance in life evaluation (R^2^ = 0.49) (S3 Table). The final model included 10 county attributes from the demographic, social and economic, clinical care, and physical environment categories, and together, these ten attributes explained 80% of the variance in the LEI (Table 4). The variables with the largest contribution to R^2^ were: higher percent black race, lower percent with less than a high school diploma, and lower percent commuting by public transportation.

**Table 4:** Combined and final models of county characteristics independently associated with LEI.

County Characteristics		Combined Model						Final Model					
		Q1	Q2	Q3	Q4	Q5	Wald P-value	Q1	Q2	Q3	Q4	Q5	Wald P-value
**Demographic Factors**													
% English Only Spoken at Home		ref	-0.44	-0.50	-0.37	-0.60	0.002	ref	-0.44	-0.47	-0.37	-0.70	<0.001
% Female													
	65+ years	ref	-0.41	-0.54	-0.56	-0.80	<0.001	ref	-0.37	-0.48	-0.47	-0.63	<0.001
Race													
	% Asian (< .2, .3–1, 1–2, >2)	ref	0.10	-0.05	-0.32	—	0.058						
	% Black (≤.5, .5–2, 2–10, 10–30, >30)	ref	0.04	0.33	1.04	2.49	<0.001	ref	0.01	0.18	0.88	2.46	<0.001
% Urban		ref	0.43	0.37	0.60	0.69	0.235						
**Social and Economic Factors**													
Education													
	% 9th to 12th Grade, No Diploma	ref	-0.75	-1.03	-1.18	-1.46	<0.001	ref	-0.79	-1.08	-1.26	-1.54	<0.001
	% High School Graduate or Equivalent	ref	-0.41	-0.67	-0.60	-0.93	<0.001	ref	-0.46	-0.75	-0.71	-1.16	<0.001
	% Bachelor's Degree	ref	0.71	1.23	1.45	1.61	<0.001	ref	0.71	1.21	1.38	1.45	<0.001
Mean Household Income		ref	0.13	0.20	0.51	0.94	<0.001	ref	0.09	0.16	0.46	0.83	<0.001
**Clinical Care Factors**													
Healthcare Practitioners													
	Dentists/100k	ref	0.42	0.22	-0.01	-0.07	0.038	ref	0.44	0.23	0.02	0.01	0.049
	GPs/Specialists	ref	-0.07	-0.13	0.08	-0.06	0.799						
Acute and Long Term Care Capacity													
# Psych Hospitals (0, 1, 2+)		ref	0.07	0.06	—	—	0.848						
% Prescription Drug Spending		ref	-0.09	0.12	0.13	0.15	0.594						
Preventable Hospital Stays		ref	-0.25	-0.31	-0.37	-0.40	0.100						
Health Professions Schools													
# MD Schools (0, 1+)		ref	0.18	—	—	—	0.243						
# RN Schools with BSN Program (0, 1+)		ref	-0.03	—	—	—	0.797						
Short Term General Hospitals Utilization Rate													
	40–59% (0,1–2,2)	ref	-0.09	-0.30	—	—	0.161						
**Physical Environment Factors**													
% Commute by													
	Bicycle (0, ≤0.1, .1–1, >1)	ref	-0.30	0.03	0.31	—	0.006	ref	-0.34	0.02	0.45	—	<0.001
	Public transit (0, ≤0.5, .5–1, 1–3, >3)	ref	0.29	0.26	0.14	-0.59	<0.001	ref	0.29	0.22	0.07	-0.75	<0.001
	Work at Home (≤2, 2–4, 4–8, 8–10, >10)	ref	-0.29	-0.15	-0.19	0.04	0.415						
Housing Unit Density per Square Mile		ref	-0.28	-0.35	-0.29	-0.54	0.269						

Combined model: independent correlation coefficients when including all variables that were independently significant in their relationship with LEI in their respective category-specific model. Final model: independent correlation coefficient in a model that includes only variables significantly associated with LEI in combined model. Abbreviations: GP = General practitioner; MD = Doctor of medicine; RN = Registered nurse; BSN = Bachelor of science in nursing

## Discussion

In this nationwide study of more than 300,000 adults and more than 75 attributes of the counties in which they reside, we identified twelve county factors that were independently associated with a comprehensive, multi-dimensional assessment of individual well-being. Together, these twelve factors explained more than ninety percent of the variance in individual well-being scores. The final set of twelve factors were from the demographic, social and economic, clinical care, and physical environment categories. The majority of these factors were also independently associated with overall life evaluation. These findings suggest that promotion of diversity as well as targeted investments in education, transportation, and primary care may lead to higher well-being of community residents, an idea worth testing. Our findings also bolster existing theoretical models that propose multi-pronged efforts to improve community factors from several categories (e.g., sociocultural, economic, political, educational, transportation, healthcare, government, religious) are necessary to promote the well-being of community members.[19, 22–25, 38, 39, 43, 44]

Our study is distinctive in its size and scale, examining a wide range of community factors and their relation to a comprehensive measure of well-being among a large sample of Americans. A second strength relates to our approach. Among existing health disparities studies, race, ethnicity, income, educational attainment, smoking status, and levels of physical activity are all highly related to each other, and thus, their unique influence on outcomes related to health and well-being are difficult to isolate.[45, 46] To better identify such relationships, we used a robust method to disentangle these community factors from each other and identify those independently associated with well-being.

Previous county- and state-level studies have reported that a higher percent of the population with a high school diploma was associated with higher aggregate life satisfaction or well-being.[17, 47] Our analysis extends this prior work in two major ways. First, we demonstrated the independent association of education with a multi-dimensional assessment of well-being at the individual level, as compared to regionally aggregated life satisfaction.[48, 49] Second, we identified independent associations across a much broader array of community factors including those related to clinical care, health behaviors, and the physical environment, in addition to other social and economic factors. These results are not only more robust but also more comprehensive.

County race composition, specifically, higher percent black, was associated with higher iWBS and LEI scores in our final models. A prior study assessing correlates of well-being at the state level similarly reported that the well-being index was positively associated with measures of inclusiveness.[47] These findings are consistent with prior studies reporting that minority status is associated with greater eudemonic well-being (i.e., an individual’s judgments about the meaning and purpose in one’s life).[11, 50] Though our findings are inconsistent with results from one study stating that racial/ethnic minorities have lower life satisfaction,[51] this negative association may be explained by higher perceived discrimination among participants.[52–55] Taken together, the results of these studies and ours suggest that greater tolerance may raise well-being for all community members.

Importantly, many of the county factors we identified as independently associated with well-being may be modifiable in the short to medium term and thus suitable targets for improving resident well-being. With two county factors related to transportation and commuting remaining in our final model for individual well-being, and the physical environment category itself explaining 64% of the variation in well-being, transportation and urban planning sectors should play a key role in designing and testing interventions that may improve the well-being of community residents. Our results clarify prior observations that long commutes and commuting alone by automobile are correlated with low levels of positive affect and life satisfaction,[17, 56, 57] and suggest potential solutions. Commuting and workplace policies such as flexibility to work from home and availability of protected bicycle lanes should be evaluated to assess their impact on well-being of residents.

Additionally, healthcare sector variables (clinical care category), when modeled alone, explained 80% of the variation in well-being, and several factors related to access to primary care and screening behaviors were independently associated with well-being in the final model. These findings extend prior observations that beyond income, perceived access to healthcare is a strong predictor of well-being. Notably, even in countries with universal healthcare coverage, perceived access to healthcare is correlated with well-being.[58] We show that objective markers of access to primary care and utilization of preventive care are, in fact, independently correlated with higher well-being. Though it is possible that access to primary care is simply a marker of an unknown confounder related to higher well-being, it is also plausible that access to healthcare mediates increased well-being by providing greater “peace of mind” and necessary support for preventive health and general self-management behaviors.[59] If this logic holds, it implies that improving access to primary care for all community members may enhance well-being.

Our analysis has limitations. As a cross-sectional analysis, it is not possible to make causal inferences. Though we included a diverse set of variables in our analysis, it is possible that associations found were due to confounding by other, unmeasured variables. With more data on well-being gathered over time, longitudinal associations may be performed in the future to assess the directionality of associations between well-being and county factors. Also, we relied on secondary data sources to identify county level attributes, which meant we were unable to capture all categories equally. For example, though we posited psychosocial variables such as social support and tolerance could influence well-being, we were unable to include them explicitly in our model because nationwide, county-level measures for these factors were unavailable. Though we were limited by the available data, our selection of candidate variables was purposeful and intended to test specific hypotheses. In addition, our well-being data were collected during the years following the economic recession. While it is possible this may limit the generalizability of our results since this is a particularly volatile economic period, all non-economic variables remaining in the final model were associated with well-being, independent of the included contemporaneous financial and economic factors. Several of these variables were unrelated to financial well-being, such as density of primary care physicians, along with child poverty rate, which is directly related to financial well-being, suggesting our results may be more broadly applicable. A final limitation is our use of county as a measure of community; because our data are at the county level, relationships that exist at the community or city-level could be missed. Nevertheless, our county-level results have important policy implications and can inform local communities in developing and testing targeted programs to enhance well-being.

Our findings highlight twelve county attributes associated with higher resident well-being. Prospective evaluation is required to assess whether changes in these county factors result in higher well-being and new models such as the CMS accountable health communities may facilitate this assessment.[19] Future research should evaluate whether improvements in education, fostering diversity, the creation of healthcare delivery models that support preventive care, and the development of environmental infrastructure that supports physical activity actually improve the well-being of community members over time. Additionally, because prior work has shown that state-level measures of inclusiveness and social tolerance are correlated with greater well-being,[47] we encourage policymakers and public health officials to include more psychosocial assessments in population surveys to provide necessary data for studies that examine these factors’ contribution to well-being. Future work should not only assess the independent influence of education, healthcare, environment, and psychosocial community factors on resident well-being, but also examine the impact of combinations of these factors.

## Supporting information

S1 Fig Conceptual model. Theoretical model with the initial 114 pre-specified county factors within six categories postulated to influence various domains of resident well-being. Italicized text denotes factors that were excluded from the study due to insufficient county level data from 2010–2012.
(PDF)

S1 Table Descriptions of Gallup-Sharecare (previously Gallup-Healthways) Well-being Index domains and survey items [22]. (DOCX)

S2 Table Mean resident life evaluation index (LEI) scores across quintiles of 77 county factors. Each factor was categorized by equally distributed quintiles, unless noted in parentheses. Bivariate associations for each county factor with resident well-being were tested and level of significance is noted by the Wald P-value.
(DOCX)

S3 Table Category-specific models for the life evaluation index (LEI). Correlation coefficients for the association between each variable that was significantly associated with LEI in bivariate analyses, independent of other variables within the same category. Each factor was categorized by equally distributed quintiles, unless noted in parentheses. P-value reported is the Wald P-value for trend across quintiles. R^2^ is the amount of variance in resident well-being explained by all factors within each category.
(DOCX)
