# Supplementary material for: Identifying county characteristics associated with resident well-being: A population based study
Source: PLoS One. 2018 May 23;13(5):e0196720. doi: 10.1371/journal.pone.0196720 (PMC5965855; doi:10.1371/journal.pone.0196720)
Supplement: S1 Fig — Theoretical model with the initial 114 pre-specified county factors within six categories postulated to influence various domains of resident well-being. Italicized text denotes factors that were excluded from the study due to insufficient county level data from 2010–2012. (PDF) [file pone.0196720.s001.pdf]

Social & Economic (23 factors)

- Education
  - Educational attainment
  - Early childhood education
  - Nursery school enrollment
  - Kindergarten enrollment
  - *University presence*
  - *Quality of schools*
  - *Library access*
- Economy
  - Income inequality
  - Median income
  - Poverty
  - Child poverty
  - Unemployment
  - *Industry presence*
- Family structure
  - Never married
  - Divorce rate
  - *Single-parent households*
  - Household size
- Safety
  - Violent crime rate
  - *Incarceration rate*
  - *Quality of policing*
- Community engagement
  - *Number of community events*
  - *Number of social clubs (e.g., 4H, Rotary, Scouts)*
  - *Voting rates*

Clinical Care (31 factors)

- Access to healthcare
  - Primary care
  - Specialty care
  - Federally qualified health centers
  - Dentists
  - *Optometrists*
  - Emergency department use
  - *Pharmacies*
  - Uninsured
  - Medicaid
  - Hospitals
  - Hospital beds (# and % occupied)
  - Nursing home beds
  - Psych hospitals
  - Preventable hospitalization rate
- Health professions training programs
  - Medical schools (MD/DO)
  - Dentistry schools
  - Nursing schools
  - Optometry schools
  - Pharmacy schools
- Preventive Care
  - A1c screening
  - Mammography
- Money spent on medical services
- Prescription drug spending
- *Practice patterns*
- *Healthcare quality*
- *Health department*

Health Behaviors (18 factors)

- Diet/nutrition
  - Eating out
  - Spending on fresh produce
  - *Fruit/veg intake*
  - Fast food presence
  - *Food availability*
  - Food access
  - *Nutrition reporting*
  - *Trans-fat ban*
  - *Sodium policies*
- *Helmet laws*
- *Alcohol use disorder*
- *Illicit drug use*
- *Smoking rates*
- *Smoking laws*
- *Physical activity rates*
- Access to recreation facilities

Physical Environment (14 factors)

- Public transport
- Commuting mode
- Farming community
- Housing density
- Water quality
- *Green space*
- *Walkability*
- *Bike paths*
- *Highway proximity*
- *Zoning laws*
- *Aesthetics*
- Toxic waste sites
- Pollution
  - Air quality
- Fine particulate matter)

Demographic (11 factors)

- Age
- Race
- Gender
- Language
- Migration
- Urban
- Rural
- Retirement destination
- *Segregation*
- *Political leaning*
- Population density

Psychosocial (17 factors)

- *Collective efficacy*
- *Neighborhood trust*
- *Government trust*
- *Religiosity*
- *Social network*
- *Social support*
- *Childhood trauma*
- *Acute stress*
- *Chronic stress*
- *Emotional intelligence*
- *Emotional support*
- *Family structure*
- *Perceived discrimination*
- *Optimism/hope*
- *Religiosity*
- *Social capacity*
- *Social cohesion*

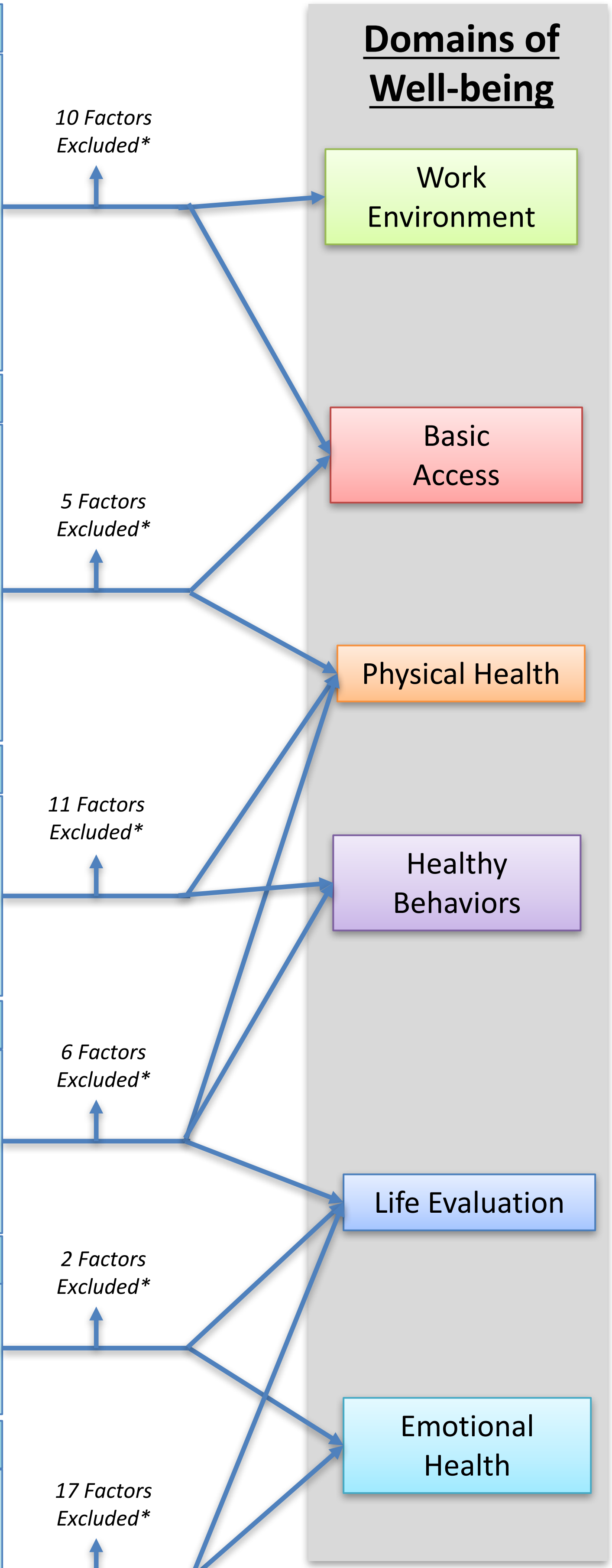

*Data not available for all counties for these variables during the years of our study period
