# Supplementary material for: Identifying county characteristics associated with resident well-being: A population based study
Source: PLoS One. 2018 May 23;13(5):e0196720. doi: 10.1371/journal.pone.0196720 (PMC5965855; doi:10.1371/journal.pone.0196720)
Supplement: S1 Table — (DOCX) [file pone.0196720.s002.docx]

**Supplement Table 1:**

| **Domain** | **Survey Item Descriptions** |
| --- | --- |
| Physical Health | Sick days in the past month |
|  | Disease burden |
|  | Health problems that get in the way of normal activities |
|  | Obesity |
|  | Feeling well-rested |
|  | Daily energy |
|  | Daily colds |
|  | Daily flu |
|  | Daily headaches |
| Emotional Health | Smiling or laughter |
|  | Learning or doing something interesting |
|  | Being treated with respect |
|  | Enjoyment |
|  | Happiness |
|  | Worry |
|  | Sadness |
|  | Anger |
|  | Stress |
|  | Diagnosed with depression |
| Life Evaluation | Imagining a ladder with steps numbered 0 to 10 where “0” represents the worst possible life and “10” represents the best possible life, on which step do you stand at this time? |
|  | On which step will you stand 5 years from now? |
| Health Behaviors | Do you smoke? |
|  | Did you eat healthy yesterday? |
|  | Weekly consumption of fruits and vegetables |
|  | Weekly exercise frequency |
| Work Environment | Are you satisfied or dissatisfied with your job or the work you do? |
|  | At work, do you get to use your strengths to do what you do best every day, or not? |
|  | Does your supervisor at work treat you more like he or she is your boss or your partner? |
|  | Does your supervisor always create an environment that is trusting and open or not? |
| Basic Access | Satisfaction with community or area |
|  | Area getting better as a place to live |
|  | Clean water |
|  | Medicine |
|  | Safe place to exercise |
|  | Affordable fruits and vegetables |
|  | Feel safe walking alone at night |
|  | Enough money for food |
|  | Enough money for shelter |
|  | Enough money for healthcare |
|  | Visited a dentist recently |
|  | Have a doctor |
|  | Have health insurance |
