# Supplementary material for: Identifying county characteristics associated with resident well-being: A population based study
Source: PLoS One. 2018 May 23;13(5):e0196720. doi: 10.1371/journal.pone.0196720 (PMC5965855; doi:10.1371/journal.pone.0196720)
Supplement: S2 Table — Each factor was categorized by equally distributed quintiles, unless noted in parentheses. Bivariate associations for each county factor with resident well-being were tested and level of significance is noted by the Wald P-value. (DOCX) [file pone.0196720.s003.docx]

**Appendix Table 1.** Mean resident life evaluation index (LEI) scores across quintiles of 77 county factors. Each factor was categorized by equally distributed quintiles, unless noted in parentheses. Bivariate associations for each county factor with resident well-being were tested and level of significance is noted by the Wald P-value.

| **County Factors** | | | | | **Q1** | **Q2** | **Q3** | **Q4** | **Q5** | **P-value** | **R^2^** |
| --- | --- | --- | --- | --- | --- | --- | --- | --- | --- | --- | --- |
| **Demographic Factors** | | | | | | | | | | | |
| % English Only Spoken at Home | | | | | 73.2 | 72.5 | 71.6 | 71.0 | 69.7 | <0.001 | 0.30 |
| % Female | | | | |  |  |  |  |  |  |  |
|  | Total | | | | 71.5 | 72.4 | 72.3 | 72.5 | 72.7 | <0.001 | 0.05 |
|  | <= 15 years | | | | 71.5 | 71.9 | 72.6 | 72.7 | 73.2 | <0.001 | 0.06 |
|  | <= 19 years | | | | 71.5 | 71.9 | 72.3 | 72.9 | 72.9 | <0.001 | 0.09 |
|  | <= 24 years | | | | 71.6 | 71.7 | 71.7 | 72.1 | 73.2 | <0.001 | 0.11 |
|  | <= 44 years | | | | 70.8 | 70.9 | 71.1 | 71.8 | 73.4 | <0.001 | 0.29 |
|  | <= 64 years | | | | 73.4 | 72.7 | 71.9 | 71.8 | 71.5 | <0.001 | 0.09 |
|  | 65+ years | | | | 73.5 | 72.3 | 71.6 | 70.6 | 70.8 | <0.001 | 0.31 |
| % Moved From a Different County | | | | | 72.2 | 72.2 | 72.6 | 72.6 | 73.3 | <0.001 | 0.08 |
| Population Density | | | | | 71.6 | 70.9 | 70.8 | 71.6 | 73.0 | <0.001 | 0.23 |
| Race | | | | |  |  |  |  |  |  |  |
|  | % Asian (.2, .3-1, 1-2, >2) | | | | 70.1 | 71.6 | 72.8 | 73.3 | -- | <0.001 | 0.55 |
|  | % Black (<.5, .5-2, 2-10, 10-30, >30) | | | | 70.8 | 71.8 | 72.4 | 73.0 | 73.9 | <0.001 | 0.46 |
|  | % White | | | | 73.5 | 72.6 | 71.9 | 71.0 | 69.9 | <0.001 | 0.30 |
| Retirement Destination (No, Yes) | | | | | 72.5 | 72.0 | -- | -- | -- | 0.330 | 0.42 |
| % Rural (≤5, 6-30) | | | | | 72.8 | 70.4 | -- | -- | -- | <0.001 | 0.50 |
| % Urban | | | | | 70.2 | 70.2 | 70.8 | 71.7 | 73.1 | <0.001 | 0.37 |
| **Social and Economic Factors** | | | | | | | | | | | |
| Education | | | | |  |  |  |  |  |  |  |
|  | % Less Than 9th Grade | | | | 72.6 | 72.3 | 72.6 | 72.6 | 72.3 | <0.001 | 0.06 |
|  | % 9th to 12th Grade, No Diploma | | | | 73.4 | 72.6 | 72.2 | 71.5 | 71.0 | <0.001 | 0.31 |
|  | % High School Graduate or Equivalent | | | | 73.5 | 72.0 | 71.2 | 70.7 | 69.6 | <0.001 | 0.55 |
|  | % Some College, No Degree | | | | 72.3 | 72.4 | 72.8 | 72.6 | 72.3 | <0.001 | 0.01 |
|  | % Associate's Degree | | | | 72.8 | 72.8 | 72.6 | 72.2 | 72.0 | 0.165 | 0.00 |
|  | % Bachelor's Degree | | | | 69.4 | 70.4 | 71.4 | 72.1 | 73.4 | <0.001 | 0.57 |
|  | % Graduate or Professional Degree | | | | 70.0 | 70.3 | 70.7 | 72.0 | 73.3 | <0.001 | 0.51 |
| GINI Coefficient | | | | | 72.1 | 72.2 | 71.7 | 72.6 | 73.2 | <0.001 | 0.06 |
| Marital Status | | | | |  |  |  |  |  |  |  |
|  | | % Never Married | | | 70.7 | 71.2 | 71.7 | 72.6 | 73.2 | <0.001 | 0.15 |
|  | | % Divorced | | | 73.3 | 72.7 | 72.4 | 71.9 | 71.2 | <0.001 | 0.12 |
| Mean Household Income | | | | | 70.7 | 71.0 | 71.3 | 72.5 | 73.4 | <0.001 | 0.40 |
| Median Household Size | | | | | 72.0 | 71.9 | 72.1 | 72.2 | 73.3 | <0.001 | 0.08 |
| Poverty | | | | |  |  |  |  |  |  |  |
|  | | | % Children in Poverty | | 73.2 | 72.2 | 72.1 | 72.1 | 71.9 | <0.001 | 0.17 |
|  | | | % Persons in Poverty | | 73.0 | 72.5 | 72.2 | 72.3 | 72.0 | <0.001 | 0.10 |
| % School Enrollment | | | | |  |  |  |  |  |  |  |
|  | Nursery School, Preschool | | | | 71.7 | 72.3 | 72.7 | 72.8 | 72.5 | <0.001 | 0.04 |
|  | Kindergarten | | | | 72.4 | 72.7 | 72.7 | 72.2 | 71.1 | <0.001 | 0.05 |
| % Single Parent Households | | | | | 72.7 | 72.3 | 72.2 | 72.2 | 72.9 | <0.001 | 0.02 |
| % Unemployed | | | | | 73.4 | 73.0 | 72.4 | 72.0 | 71.4 | <0.001 | 0.19 |
| Violent Crime Rate | | | | | 72.0 | 72.0 | 72.3 | 72.5 | 72.9 | 0.003 | 0.10 |
| **Clinical Care Factors** | | | | | | | | | | | |
| % Diabetes with HbA1c Test | | | | | 72.6 | 72.7 | 72.5 | 72.3 | 72.0 | 0.051 | 0.01 |
| ED Visits/100k | | | | | 71.1 | 72.9 | 72.7 | 72.1 | 72.1 | <0.001 | 0.06 |
| # Federally Qualified Health Centers (0,1,2+) | | | | | 71.7 | 72.0 | 72.8 | -- | -- | <0.001 | 0.44 |
| Healthcare Practitioners | | | | |  |  |  |  |  |  |  |
|  | Dentists/100k | | | | 70.3 | 71.1 | 71.6 | 72.3 | 73.1 | <0.001 | 0.27 |
|  | GPs/100k | | | | 71.3 | 72.1 | 72.7 | 72.6 | 72.7 | <0.001 | 0.08 |
|  | GPs/Specialists | | | | 73.0 | 72.0 | 71.1 | 71.0 | 70.6 | <0.001 | 0.21 |
| % Health Spending | | | | | 73.0 | 72.7 | 71.5 | 71.1 | 70.6 | <0.001 | 0.23 |
| Acute and Long Term Care Capacity | | | | |  |  |  |  |  |  |  |
|  | # Hospitals (0, 1, 2, 3-4, 5-10, 11+) | | | 70.9 | 71.2 | 72.1 | 72.2 | 72.9 | 73.3 | <0.001 | 0.47 |
|  | # Hospital Beds | | | | 70.9 | 70.8 | 71.0 | 71.7 | 72.9 | <0.001 | 0.18 |
|  | # NH Beds (0, 50, 51-100, 101+) | | | | 72.1 | 72.8 | 72.5 | 73.1 | -- | <0.001 | 0.43 |
|  | # Psych Hospitals (0, 1, 2+) | | | | 72.0 | 73.1 | 73.4 | -- | -- | <0.001 | 0.44 |
| % Mammography | | | | | 71.7 | 72.3 | 72.6 | 72.6 | 72.5 | <0.001 | 0.08 |
| % Medicaid | | | | | 73.3 | 72.6 | 72.2 | 72.0 | 72.1 | <0.001 | 0.18 |
| % Prescription Drug Spending | | | | | 73.0 | 72.7 | 71.9 | 70.9 | 70.5 | <0.001 | 0.21 |
| Preventable Hospital Stays | | | | | 73.1 | 72.7 | 72.0 | 71.4 | 70.3 | <0.001 | 0.28 |
| Health Professions Schools | | | | |  |  |  |  |  |  |  |
|  | # DDS Schools (0, 1+) | | | | 72.3 | 73.4 | -- | -- | -- | <0.001 | 0.43 |
|  | # DO Schools (0, 1+) | | | | 72.4 | 72.9 | -- | -- | -- | 0.275 | 0.42 |
|  | # MD Schools (0, 1+) | | | | 72.1 | 73.6 | -- | -- | -- | <0.001 | 0.44 |
|  | # Optometry Schools (0, 1+) | | | | 72.3 | 74.1 | -- | -- | -- | 0.002 | 0.42 |
|  | # Pharmacy Schools (0, 1+) | | | | 72.2 | 73.3 | -- | -- | -- | <0.001 | 0.43 |
|  | # RN Schools with BSN Program (0, 1+) | | | | 71.8 | 73.2 | -- | -- | -- | <0.001 | 0.45 |
| Short Term General Hospitals Utilization Rate | | | | |  |  |  |  |  |  |  |
|  | 00 - 39% (0,1-2,2) | | | | 72.3 | 72.5 | 73.9 | -- | -- | 0.012 | 0.42 |
|  | 40 - 59% (0,1-2,2) | | | | 72.1 | 72.4 | 73.1 | -- | -- | <0.001 | 0.43 |
|  | 60 - 79% (0,1-2,2) | | | | 71.5 | 72.3 | 73.3 | -- | -- | <0.001 | 0.46 |
|  | 80+% (0,1-2,2) | | | | 72.1 | 73.0 | 73.3 | -- | -- | <0.001 | 0.43 |
| % Uninsured Adults | | | | | 72.1 | 72.2 | 72.8 | 72.6 | 72.8 | 0.028 | 0.01 |
| **Physical Environment Factors** | | | | | | | | | | | |
| % Commute by | | | | |  |  |  |  |  |  |  |
|  | Bicycle (0, ≤0.1, .1-1, >1) | | | | 70.7 | 71.9 | 72.6 | 73.5 | -- | <0.001 | 0.47 |
|  | Car, Truck, or Van | | | | 73.4 | 72.5 | 72.3 | 71.9 | 71.3 | <0.001 | 0.14 |
|  | Public transit (0, ≤0.5, .5-1, 1-3, >3) | | | | 70.3 | 71.4 | 72.2 | 72.9 | 73.3 | <0.001 | 0.48 |
|  | Walk (≤1, 1-3, 3-5, 5-10, >10) | | | | 72.0 | 72.5 | 72.3 | 72.7 | 74.3 | 0.003 | 0.42 |
|  | Work at Home (≤2, 2-4, 4-8, 8-10, >10) | | | | 71.3 | 72.2 | 72.8 | 72.4 | 72.6 | <0.001 | 0.44 |
| Daily Fine Particulate Matter | | | | | 73.0 | 72.4 | 72.1 | 72.7 | 71.8 | <0.001 | 0.07 |
| Farming Community (No, Yes) | | | | | 72.5 | 71.2 | -- | -- | -- | 0.044 | 0.42 |
| % Good Air Quality Days | | | | | 73.0 | 73.1 | 72.4 | 72.2 | 72.0 | 0.002 | 0.32 |
| Housing Unit Density per Square Mile | | | | | 71.6 | 71.0 | 70.9 | 71.5 | 73.0 | <0.001 | 0.21 |
| Number of Nearby Toxic Waste Sites (0,1,2+) | | | | | 72.2 | 72.5 | 73.3 | -- | -- | <0.001 | 0.43 |
| % Water Violation (1-5, 6-10, >10) | | | | | 72.4 | 72.6 | 72.4 | 71.9 | -- | <0.001 | 0.43 |
| **Health Behaviors Factors** | | | | | | | | | | | |
| % Food Out Spending | | | | | 72.3 | 72.1 | 71.3 | 72.1 | 73.2 | <0.001 | 0.12 |
| % Fruit/Veg Spending | | | | | 72.8 | 72.3 | 72.2 | 72.1 | 72.7 | <0.001 | 0.03 |
| % People with Limited Access to Health Foods | | | | | 71.2 | 72.0 | 72.5 | 72.7 | 72.6 | <0.001 | 0.05 |
| Recreational Facilities/100k | | | | | 70.3 | 71.6 | 72.4 | 72.5 | 73.0 | <0.001 | 0.16 |
| % Restaurants that Serve Fast Food | | | | | 71.1 | 71.8 | 72.5 | 72.9 | 72.5 | <0.001 | 0.05 |

Abbreviations: HbA1c = Hemoglobin A1c; ED = Emergency department; GP = General practitioner; NH = Nursing home; RN = Registered nurse; BSN = Bachelor of science in nursing; DDS = Doctor of dental surgery; DO = Doctor of osteopathy; MD = Doctor of medicine
