# Supplementary material for: Identifying county characteristics associated with resident well-being: A population based study
Source: PLoS One. 2018 May 23;13(5):e0196720. doi: 10.1371/journal.pone.0196720 (PMC5965855; doi:10.1371/journal.pone.0196720)
Supplement: S3 Table — Correlation coefficients for the association between each variable that was significantly associated with LEI in bivariate analyses, independent of other variables within the same category. Each factor was categorized by equally distributed quintiles, unless noted in parentheses. P-value reported is the Wald P-value for trend across quintiles. R2 is the amount of variance in resident well-being explained by all factors within each category. (DOCX) [file pone.0196720.s004.docx]

**Appendix Table 2.** Category-specific models: Correlation coefficients for the association between each variable that was significantly associated with LEI in bivariate analyses, independent of other variables within the same category. Each factor was categorized by equally distributed quintiles, unless noted in parentheses. P-value reported is the Wald P-value for trend across quintiles. R^2^ is the amount of variance in resident well-being explained by all factors within each category.

| **Community Factors** | | | **Q1** | **Q2** | **Q3** | **Q4** | **Q5** | **P-value** | **R2** |
| --- | --- | --- | --- | --- | --- | --- | --- | --- | --- |
| **Demographic Factors** | | | | | | | | | 0.548 |
| % English Only Spoken at Home | | | ref | -0.06 | -0.13 | -0.07 | -0.74 | 0.030 |  |
| % Female | | |  |  |  |  |  |  |  |
|  | <= 44 years | | ref | -0.14 | -0.28 | -0.35 | -0.23 | 0.622 |  |
|  | 65+ years | | ref | -0.54 | -0.67 | -0.82 | -0.72 | <0.001 |  |
| Population Density | | | ref | -0.50 | -0.72 | -0.57 | -0.61 | 0.133 |  |
| Race | | |  |  |  |  |  |  |  |
|  | % Asian (<.2, .2-1, 1-2, >2) | | ref | 0.70 | 1.11 | 1.26 | -- | <0.001 |  |
|  | % Black (<.5, .5-2, 2-10, 10-30, >30) | | ref | -0.41 | -0.73 | -0.19 | 0.82 | <0.001 |  |
|  | % White | | ref | -0.08 | -0.05 | -0.22 | -0.65 | 0.190 |  |
| % Rural (≤5, 6-30) | | | ref | -0.02 | -- | -- | -- | 0.943 |  |
| % Urban | | | ref | 0.13 | 0.36 | 0.71 | 1.12 | 0.016 |  |
| **Social and Economic Factors** | | | | | | | | | 0.669 |
| Education | | |  |  |  |  |  |  |  |
|  | % 9th to 12th Grade, No Diploma | | ref | -0.42 | -0.43 | -0.19 | 0.09 | 0.001 |  |
|  | % High School Graduate or Equivalent | | ref | -0.71 | -1.06 | -1.18 | -1.72 | <0.001 |  |
|  | % Bachelor's Degree | | ref | 0.83 | 1.60 | 1.82 | 2.14 | <0.001 |  |
| Mean Household Income | | | ref | 0.19 | 0.22 | 0.57 | 0.81 | <0.001 |  |
| **Clinical Care Factors** | | | | | | | | | 0.493 |
| # Federally Qualified Health Centers (0,1,2+) | | | ref | 0.00 | -0.15 | -- | -- | 0.441 |  |
| Healthcare Practitioners | | |  |  |  |  |  |  |  |
|  | Dentists/100k | | ref | 0.42 | 0.29 | 0.37 | 0.66 | 0.032 |  |
|  | GPs/Specialists | | ref | -0.47 | -0.90 | -0.69 | -0.84 | <0.001 |  |
| Acute and Long Term Care Capacity | | |  |  |  |  |  |  |  |
|  | # Hospitals (0, 1, 2, 3-4, 5-10, 11+) | ref | 0.42 | 0.66 | 0.52 | 0.60 | 0.67 | 0.314 |  |
|  | # NH Beds (0, 50, 51-100, 101+) | | ref | 0.13 | -0.13 | -0.05 | -- | 0.692 |  |
|  | # Psych Hospitals (0, 1, 2+) | | ref | 0.37 | 0.21 | -- | -- | 0.093 |  |
| % Prescription Drug Spending | | | ref | -0.19 | -0.42 | -0.79 | -0.88 | <0.001 |  |
| Preventable Hospital Stays | | | ref | -0.43 | -0.84 | -1.20 | -1.56 | <0.001 |  |
| Health Professions Schools | | |  |  |  |  |  |  |  |
|  | # DDS Schools (0, 1+) | | ref | -0.19 | -- | -- | -- | 0.522 |  |
|  | # MD Schools (0, 1+) | | ref | 0.53 | -- | -- | -- | 0.026 |  |
|  | # Optometry Schools (0, 1+) | | ref | 0.52 | -- | -- | -- | 0.197 |  |
|  | # Pharmacy Schools (0, 1+) | | ref | -0.03 | -- | -- | -- | 0.875 |  |
|  | # RN Schools with BSN Program (0, 1+) | | ref | 0.30 | -- | -- | -- | 0.030 |  |
| Short Term General Hospitals Utilization Rate | | |  |  |  |  |  |  |  |
| 0 - 39% (0,1-2,2) | | | ref | 0.06 | 0.75 | -- | -- | 0.274 |  |
| 40 - 59% (0,1-2,2) | | | ref | -0.25 | -0.55 | -- | -- | 0.053 |  |
| 60 - 79% (0,1-2,2) | | | ref | -0.15 | -0.09 | -- | -- | 0.590 |  |
| 80+% (0,1-2,2) | | | ref | -0.26 | -0.56 | -- | -- | 0.131 |  |
| **Physical Environment Factors** | | | | | | | | | 0.540 |
| % Commute by | | |  |  |  |  |  |  |  |
|  | Bicycle (0, ≤0.1, .1-1, >1) | | ref | 0.48 | 1.04 | 1.67 | -- | <0.001 |  |
|  | Public transit (0, ≤0.5, .5-1, 1-3, >3) | | ref | 1.00 | 1.32 | 1.52 | 1.51 | 0.004 |  |
|  | Walk (≤1, 1-3, 3-5, 5-10, >10) | | ref | 0.00 | -0.40 | -0.27 | -0.09 | 0.178 |  |
|  | Work at Home (≤2, 2-4, 4-8, 8-10, >10) | | ref | 0.01 | 0.58 | 0.56 | 1.39 | <0.001 |  |
| Farming Community (No, Yes) | | | ref | -0.01 | -- | -- | -- | 0.984 |  |
| % Good Air Quality Days | | | ref | 0.25 | -0.10 | -0.01 | 0.10 | 0.411 |  |
| Housing Unit Density per Square Mile | | | ref | -0.45 | -0.32 | 0.28 | 0.85 | <0.001 |  |
| Number of Nearby Toxic Waste Sites (0,1,2+) | | | ref | -0.16 | -0.19 | -- | -- | 0.454 |  |
| % Water Violation (1-5, 6-10, >10) | | | ref | -0.23 | -0.13 | -0.33 | -- | 0.285 |  |
|  | | |  |  |  |  |  |  |  |

Abbreviations: GP = General practitioner; RN = Registered nurse; BSN = Bachelor of science in nursing
